# *Tnfrsf12a*-Mediated Atherosclerosis Signaling and Inflammatory Response as a Common Protection Mechanism of Shuxuening Injection Against Both Myocardial and Cerebral Ischemia-Reperfusion Injuries

**DOI:** 10.3389/fphar.2018.00312

**Published:** 2018-04-06

**Authors:** Ming Lyu, Ying Cui, Tiechan Zhao, Zhaochen Ning, Jie Ren, Xingpiao Jin, Guanwei Fan, Yan Zhu

**Affiliations:** ^1^Tianjin State Key Laboratory of Modern Chinese Medicine, Tianjin University of Traditional Chinese Medicine, Tianjin, China; ^2^Research and Development Center of TCM, Tianjin International Joint Academy of Biomedicine, Tianjin, China; ^3^First Teaching Hospital of Tianjin University of Traditional Chinese Medicine, Tianjin, China

**Keywords:** Shuxuening injection, myocardial ischemia-reperfusion, cerebral ischemia-reperfusion, atherosclerosis signaling, inflammatory response, *TNFRSF12A*

## Abstract

Shuxuening injection (SXNI) is a widely prescribed herbal medicine of Ginkgo biloba extract (EGB) for cerebral and cardiovascular diseases in China. However, its curative effects on ischemic stroke and heart diseases and the underlying mechanisms remain unknown. Taking an integrated approach of RNA-seq and network pharmacology analysis, we compared transcriptome profiles of brain and heart ischemia reperfusion injury in C57BL/6J mice to identify common and differential target genes by SXNI. Models for myocardial ischemia reperfusion injury (MIRI) by ligating left anterior descending coronary artery (LAD) for 30 min ischemia and 24 h reperfusion and cerebral ischemia reperfusion injury (CIRI) by middle cerebral artery occlusion (MCAO) for 90 min ischemia and 24 h reperfusion were employed to identify the common mechanisms of SXNI on both cerebral and myocardial ischemia reperfusion. In the CIRI model, ischemic infarct volume was markedly decreased after pre-treatment with SXNI at 0.5, 2.5, and 12.5 mL/kg. In the MIRI model, pre-treatment with SXNI at 2.5 and 12.5 mL/kg improved cardiac function and coronary blood flow and decreased myocardial infarction area. Besides, SXNI at 2.5 mL/kg also markedly reduced the levels of LDH, AST, CK-MB, and CK in serum. RNA-seq analysis identified 329 differentially expressed genes (DEGs) in brain and 94 DEGs in heart after SXNI treatment in CIRI or MIRI models, respectively. Core analysis by Ingenuity Pathway Analysis (IPA) revealed that atherosclerosis signaling and inflammatory response were top-ranked in the target profiles for both CIRI and MIRI after pre-treatment with SXNI. Specifically, *Tnfrsf12a* was recognized as an important common target, and was regulated by SXNI in CIRI and MIRI. In conclusion, our study showed that SXNI effectively protects brain and heart from I/R injuries via a common *Tnfrsf12a*-mediated pathway involving atherosclerosis signaling and inflammatory response. It provides a novel knowledge of active ingredients of Ginkgo biloba on cardio-cerebral vascular diseases in future clinical application.

## Introduction

Both ischemic myocardial infarction and ischemic stroke are the leading causes of disability and death of cardio-cerebral vascular diseases (Benjamin et al., 2017). One of the causes is ischemia and reperfusion–induced tissue or organ injury, leading to morbidity and mortality in a wide-range of pathologies (Eltzschig and Eckle, 2011). Diverse pathological factors, such as inflammation (Li et al., 2012), oxidative stress (Lakshmi et al., 2009), and apoptosis (Liou et al., 2011) compose the underlying pathological mechanism of MIRI. Although restoring the blood reperfusion as soon as possible will contribute to attenuate cerebral ischemic injury in clinic, the following reperfusion can also cause cerebral edema, brain hemorrhage, and neuronal death, resulting in brain injury (Phillis, 1994). Multiple mechanisms are involved in cerebral ischemia/reperfusion injury (CIRI), including free radical-induced inflammation, energy metabolism disorder, excitotoxicity, abnormal opening of blood–brain barrier, oxidative stress, and calcium overload (Shibata et al., 2001; Lakshmi et al., 2009; Chen et al., 2011; Cook et al., 2012; Liu Y. et al., 2013; Orsu et al., 2013). Traditional Chinese medicine (TCM) has been widely prescribed in clinics in China and some Asian countries for more than 2000 years. Studies over the past decades revealed that certain TCM and their major active ingredients have a protective and therapeutic effect on I/R-induced injury in brain and heart (Han et al., 2017).

Ginkgo biloba extracts (EGB, especially EGb761 and EGb50) are among the most popular herbal dietary supplements, frequently applied for multiple cardio-cerebral vascular indications (Zhou et al., 2004). A recent search of PubMed with the keyword combinations of “Ginkgo biloba AND brain” or “Ginkgo biloba AND heart” yielded 579 or 145 reports, respectively. For brain-related diseases, EGB is reported to have a beneficial effect on acute cerebral infarction (Wu et al., 2015), chronic cerebral hypoperfusion (Kim et al., 2016), brain damage (Tulsulkar et al., 2016), ischemic stroke (Calapai et al., 2000; Nada et al., 2014), hippocampal neuronal loss (Rocher et al., 2011; Tulsulkar and Shah, 2013), cognitive impairment (Belviranli and Okudan, 2015), memory deficit (Oliveira et al., 2009; Abdel-Wahab and Abd El-Aziz, 2012), dementia (DeKosky et al., 2008), Parkinson’s disease (Tanaka et al., 2013) and Alzheimer’s disease (Shi et al., 2010; Liu et al., 2015), with its mechanisms of action such as resisting apoptosis, oxidative stress, inflammatory response, immune response and platelet activation (Kleijnen and Knipschild, 1992; Smith and Luo, 2004; Chan et al., 2007; Diamond and Bailey, 2013; Mohanta et al., 2014). In addition, EGB also exerts protective effect on heart-related diseases, including cardiac injury (Boghdady, 2013), arrhythmia (Zhao et al., 2013), myocardial ischemia (Wang Z. et al., 2016), myocardial infarction (Liu A. H. et al., 2013; Liu et al., 2014) and doxorubicin-induced cardiotoxicity (Naidu et al., 2002) mainly via antioxidant, anti-apoptotic, anti-inflammation, and anti-mitochondrial oxidative damage. Furthermore, numerous studies reported that EGB could attenuate both MIRI (Haramaki et al., 1994; Shen and Zhou, 1995; Liebgott et al., 2000; Kusmic et al., 2004; Schneider et al., 2009; Lu et al., 2011) and CIRI (Hu et al., 2002; Saleem et al., 2008; Zhang et al., 2012; Yang et al., 2013). The most two recent reports demonstrated that EGB could reduce MIRI via TLR-4/NF-κB signaling pathway (Tang et al., 2017) and ameliorate cardiac hypertrophy via activation of muscarinic receptors (M2)/nitric oxide (NO) pathway (Mesquita et al., 2017). The main identified pharmacological active ingredients are flavonols (kaempferol, quercetin, myricetin, apigenin, isorhamnetin, luteolin, and tamarixetin) and terpene trilactones (ginkgolide A, ginkgolide B, ginkgolide C, ginkgolide J, ginkgolide M, ginkgolide K, ginkgolide L, ginkgolide P, ginkgolide Q, and bilobalide) (van Beek and Montoro, 2009; Liao et al., 2011; Mohanta et al., 2014). Shuxuening injection (SXNI), one of the pharmaceutical preparations of EGB, is a Sino Food and Drug Administration (SFDA)-approved Chinese Materia Medica consisting of Ginkgo flavonol glycosides and Ginkgolides applied widely for ischemia stroke and coronary heart disease (CHD) in clinic in China (Zhang et al., 2009; Zhou, 2011; Guo et al., 2012; Wang et al., 2013; Luo et al., 2014; Chen H.Y. et al., 2017), which might improve critical ischemia and decrease reperfusion injury.

Moreover, an increasing number of studies began to focus on brain-heart connection (Bot and Kuiper, 2017; Chen Z. et al., 2017; Fujiu et al., 2017). In previous studies, we have explored that endothelial cell inflammation therapy might serve as a common underlying mechanism of another Chinese Materia Medica, Danhong injection (DHI), for stroke and coronary artery disease (CAD) treatment, using an integrated network pharmacology and experimental verification approach (Lyu et al., 2017). In addition, we have also reported that reinstating neuronal arginine vasopressin (AVP) level was one of the shared mechanisms contributing to protection of neuronal and cardiac cells from oxygen-glucose deprivation injury by DHI (Yang et al., 2016). Since the reported effects of Ginkgo biloba extracts on brain and heart were diverse and sometimes controversial, and the involved molecular mechanisms were not clear, we employed a comparative transcriptome method in the present study to explore the common and differential effects and the underling mechanisms of Shuxuening injection (SXNI) on both myocardial ischemia-reperfusion injury (MIRI) and cerebral ischemia-reperfusion injury (CIRI). Our new findings may shed new light on the clinical and pharmacological application for SXNI, and may facilitate a better understanding of detailed mechanisms of I/R injury in brain and heart and discovering natural product-derived agents for co-treatment of cardio-cerebral diseases.

## Materials and Methods

### Drugs and Reagents

Shuxuening injection (drug approval number: Z13020795; batch number: 15101201) was provided by China Shineway Pharmaceutical Group Ltd. (Shijiazhuang, China). The chemical content of a SXNI (5 mL/unit), containing 4.2 mg total Ginkgo flavonol glycosides and 0.7 mg Ginkgolides, is equal to 17.5 mg Ginkgo biloba leaf extract according to manufacturer’s instruction. Valsartan (drug approval number: H20040217) was purchased from Beijing Novartis Pharma Ltd. (Beijing, China). 2,2,2-Tribromoethanol was purchased from Sigma (T48402, St. Louis, MO, United States). 2,3,5-Triphenyl-2H-Tetrazolium Chloride (TTC) was purchased from Solarbio (T8170, Beijing, China). Hematoxylin and eosin (H&E) staining kit was purchased from Beyotime Biotechnology (C0105, Shanghai, China). ELISA kits for Lactate dehydrogenase (LDH), Aspartate aminotransferase (AST), Creatine kinase-MB (CK-MB) and Creatine kinase (CK) were purchased from Biosino Bio-Technology and Science Inc. (Beijing, China).

### Animals

Male C57BL/6J mice (22 ± 2 g) were purchased from Beijing Vital River Laboratory Animal Technology Co., Ltd. (Beijing, China, Certificate no.: SCXK Jing 2012-0001). This study was carried out in accordance with the recommendations in the Guidance for the Care and Use of Laboratory Animals issued by the Ministry of Science and Technology of China and the protocol approved by the Laboratory Animal Ethics Committee of Tianjin University of TCM (Permit Number: TCM-LAEC2014004). The animals were housed in cages at a temperature of 22 °C ± 2°C and humidity of 40% ± 5%, under a 12-h light/dark cycle, with free access to food and water. In the previous experiments, animals were randomized into several groups. The experimental procedures were according to the European Union (EU) adopted Directive 2010/63/EU, and all animals were administrated following the guidelines of Tianjin University of TCM Animal Research Committee (TCM-LAEC2014004).

### MIRI Model and Drug Administration

The mouse MIRI model was performed as previously described (Gao et al., 2010). Briefly, mice were anesthetized intraperitoneally with 1.5% Tribromoethanol (150 mg/kg) and then placed in a supine position. For myocardial ischemia model preparation, the chest was opened via a left thoracotomy through the intercostal space between 3 and 4 sternal rib, and the heart was exposed. A slipknot was tied around the left anterior descending (LAD) coronary artery 1–2 mm under left auricle utilizing a 7-0 silk suture. Then the thorax was closed, and as soon as spontaneous respiration was sufficient, the mice were released and allowed to recover on an electric blanket. After 30 min of ischemia, the slipknot was released and followed by 24 h of reperfusion. ST-segment elevation on an electrocardiogram monitor represented a success in MIRI model surgery. Sham-operated animals were subjected to the same surgical procedures without ligating LAD coronary artery. The mice were randomly divided into four or five groups in the following experiments:Sham, I/R, I/R+SXNI (medium dose, 2.5 mL/kg), I/R+SXNI (high dose, 12.5 mL/kg) and I/R+valsartan (20 mg/kg). SXNI were administered via tail intravenous injection within 10 min after the beginning of ischemia, and valsartan via gavage pre-administration for 1 week. Sham and I/R groups were intravenous administered with 0.9% normal saline. No difference was observed in surgical mortality among groups investigated. At 24 h hours after reperfusion, the heart was quickly excised, frozen at -70°C, and the ventricular tissue below the ligation was cut into four slices perpendicular to the long axis of the heart. The heart sections were then incubated in a 24-well culture plate with 1% TTC solution at 37°C for 15 min, respectively. After staining by TTC, red parts in the heart indicated ischemic but viable tissue, while pale areas represented infarcted myocardium. Images were digitally captured using a microscope (S8APO, Leica, Solms, Germany), and infarct size areas were determined with planimetry software (Image J; National Institutes of Health, Bethesda, MD, United States). The size of infarction area was expressed as percentage of the ventricle area.

### CIRI Model and Drug Administration

Anesthesia was induced with 2% isoflurane inhalation and then maintained with 1.5% isoflurane in 70% nitrous oxide/30% oxygen after endotracheal intubation and mechanical ventilation by a small animal respirator (RWD, Inc., China). After a midline incision at the neck, the right carotid bifurcation was exposed, and the following external carotid artery (ECA) branches were cut after electrocoagulation: the occipital, the cranial thyroid, the pterygopalatine artery, and the ascending pharyngeal artery. After occlusion of the common carotid artery (CCA) by a micro-clip, the right ECA was ligated and cut distally to the cranial thyroid artery. A silicone-coated 4-0 nylon monofilament (Prolene, Ethicon) was introduced into the ECA and gently advanced through the internal carotid artery (ICA) until its tip occluded the origin of the middle cerebral artery (MCA). Through this, local cortical blood flow in the right MCA territory dropped to 20% of baseline. Then, MCA occlusion (MCAO) mice were then divided into five groups: model (saline), SXNI (low dose, 0.5 mL/kg), SXNI (medium dose, 2.5 mL/kg), SXNI (high dose, 12.5 mL/kg), and edaravone (9 ml/kg). Sham operated mice were processed identically except for MCAO which served as normal control. Mice were treated via intravenous injection once after 90 min of ischemia. The endovascular suture remained in place until reperfusion was allowed by withdrawal of the filament and removal of the micro-clip at the CCA after 120 min of ischemia. After 24 h, ratios of infarct volumes were measured and expressed as a percentage. All mice were euthanized at 24 h after ischemia. Then, the brains were removed and freshly cut (eight 1 mm-thick coronal slices) in a brain matrix device. The brain slices were stained with 2% solution of TTC in 0.9% normal saline at 37°C for 30 min in the dark. After staining, sections were again washed twice in normal saline and fixed with 4% paraformaldehyde for 1 h at room temperature. Ischemic infarct volumes were assessed using Image J analysis.

### Echocardiographic Measurement

Cardiac left ventricular function and coronary blood flow were assessed non-invasively at 18 h after MIRI using an ultra-high resolution small animal ultrasound Vevo 2100 Imaging System (VisualSonics, Toronto, ON, Canada) equipped with a 30 MHz transducer (Chen et al., 2016; Li et al., 2016). The mice were anesthetized using 1.5–2.0% isoflurane in O_2_ gas. When fully anesthetized, each mouse was transferred to dorsal recumbency and placed on a heated imaging platform. The following parameters as indicators of cardiac function were measured by M-mode and Color Doppler mode: left ventricular (LV) ejection fraction (EF), LV fractional shortening (FS), cardiac output (CO), stroke volume, LV internal dimensions at diastole (LVIDd), LV internal diameter systole (LVIDs), LV posterior wall diastole (LVPWd), LV posterior wall systole (LVPWs), LV systole volume (LV Vols), LV diastole volume (LV Vold), heart rate and LV mass, along with aortic valve (AV) peak velocity, AV peak pressure and aorta velocity-time integral mean velocity (AoV VTI).

### Measurement of Histopathological Examination and Biochemical Parameters

Myocardial damage was evaluated by measuring plasma concentration of LDH, AST, CK-MB, and CK, routine indicators for myocardial inflammation and acute myocardial infarction commonly used for clinical diagnostics. At the end of the experiment, blood was collected and serum was separated by centrifugation at 1,000 rpm for 15 min. The biochemical parameters were detected using ELISA kit by automatic biochemical analyzer (Multiskan MK3; Thermo Fisher Scientific, Waltham, MA, United States), according to the manufacturer’s instruction as previously described. After collecting the blood samples, the hearts were removed. Heart tissues were fixed in 10% paraformaldehyde solution for more than 48 h, embedded in paraffin, sliced into pieces of 5 μm thick, and stained with H&E. The histopathological changes were detected by optical microscope (Moticam Pro 282A, Motic China Group Co., Ltd., Xiamen, China).

### Sample Preparation and Sequencing

In separate experiments, the left ventricular myocardium sections of MIRI and the brain slices from CIRI with or without SXNI treatment (medium dose) were collected as discovery cohorts for high-throughput sequencing. Total RNA of each sample was extracted with Trizol reagent (Invitrogen, CA) according to the manufacturer’s instructions. The RNA concentration, purity and integrity were separately measured using Qubit^®^ RNA Assay Kit in Qubit^®^ 2.0 Fluorometer^®^ (Life Technologies, CA, United States), the NanoPhotometer spectrophotometer (IMPLEN, CA, United States) and the RNA Nano 6000 Assay Kit of the Bioanalyzer 2100 system (Agilent Technologies, Santa Clara, CA, United States). A total amount of 3 μg RNA per sample was used as input material for the RNA sample preparations. The resulting cDNA libraries were generated using NEBNext^®^ Ultra^TM^ RNA Library Prep Kit for Illumina^®^ (NEB, United States) following manufacturer’s recommendations and the quality was assessed on the Agilent Bioanalyzer 2100 system. The clustering of the index-coded samples was performed on a cBot Cluster Generation System using TruSeq PE Cluster Kit v3-cBot-HS (Illumina) according to the manufacturer’s instructions. After cluster generation, the library preparations were sequenced on an Illumina Hiseq platform and 125 bp/150 bp paired-end reads were generated.

### High-Throughput Data Analysis

Raw data of fastq format were firstly processed and the clean data were obtained by removing reads containing adapter, reads containing ploy-N and low quality reads from raw data. All the downstream analyses were based on the clean data with high quality. The reference sequences used were genome of *Mus musculus*. Clean reads were, respectively, aligned to the reference genome using Bowtie v2.2.3. HTSeq v0.6.1 was used to count the reads numbers mapped to each gene. And then FPKM, currently the most commonly used method for estimating gene expression levels, was calculated based on the length of the gene and reads count mapped to this gene. Differential expression analysis of two groups was performed using the DESeq R package (1.18.0). log_2_(Fold change) of 1 and *P*-value of 0.05 were set as the threshold for significantly differential expression.

### Pathway and Function Analysis of Differentially Expressed Genes (DEGs) Using Ingenuity^®^ Pathway Analysis

The gene expression data derived from RNA-Seq was used to generate core and functional analysis by Ingenuity^®^ Pathway Analysis (IPA). Genes with cutoffs of fold change ≥ 2 and *p*-Value ≤ 0.05 after SXNI treatment were set to identify and assign the molecules to the Ingenuity’s Knowledge Base. Each identifier except the novel transcripts was mapped to its corresponding object in Ingenuity’s Knowledge Base. The core and functional analysis were conducted in IPA with the following settings: Ingenuity Knowledge Base as a reference set; endogenous chemicals not included; direct and indirect relationships. Furthermore, canonical pathways and diseases and functions analysis were performed to identified significantly relevant pathways and functions which were considered for further analysis. The significance was measured in two principles: (1) the ratio of the number of molecules from the data set that map to the pathway divided by the total genes of the corresponding pathways, and (2) Fisher’s exact test was used to calculate a *p*-value determining the probability.

### RNA Extraction and RT-PCR

Total RNA samples were isolated using EasyPure1 RNA Kit (TransGen Biotech, Beijing, China) according to manufacturer’s protocols. Reverse transcription was then performed using Transcriptor First Strand cDNA Synthesis Kit (Roche, Mannheim, Germany) to obtain cDNA. A SYBR Green I-based real-time quantitative PCR was conducted to analyze the expression of target gene including tumor necrosis factor receptor superfamily member 12a (*Tnfrsf12a*), interleukin 6 (*IL-6*), collagen type III alpha 1(*Col3a1*) and phospholipase A2 group IIF (*Pla2g2f*). The relative mRNA level was determined using the comparative CT method and was normalized to the housekeeping gene glyceraldehyde-3-phosphate dehydrogenase (*GAPDH*). The primers were synthesized by the Sangon Company (Shanghai, China). Primer sequences were as follows (**Table 1**).

**Table 1:** Primer sequences.

Primer name	Primer sequence (5′–3′)
Tnfrsf12a sense	CCCCAGTACACACGGAAACAA
Tnfrsf12a antisense	CTCCCTCCCCTCCAAACATTA
IL6 sense	GCCCACCAAGAACGATAGTCA
IL6 antisense	ACCAGCATCAGTCCCAAGAAG
Col3a1 sense	AGCGGCTGAGTTTTATGACG
Col3a1 antisense	CAGGTGTAGAAGGCTGTGGG
Pla2g2f sense	TACGGCTGCTACTGCGGG
Pla2g2f antisense	GTAGACCCCAGCGGGACAT
GAPDH sense	TGGTGAAGCAGGCATCTGAG
GAPDH antisense	TGCTGTTGAAGTCGCAGGAG

### Statistical Analysis

All experiments were expressed as the mean ± SEM or mean ± SD. Statistical analysis Statistical analysis was carried out using Student’s two-tailed *t*-test for comparison between two groups and One-way analysis of variance (ANOVA) followed by Dunnett’s test the data involved three or more groups. *P* < 0.05 was defined as significant. All tests were performed using GraphPad Prism 7 software (GraphPad Software, Inc., La Jolla, CA, United States).

## Results

### SXNI Improved the Cardiac Function in MIRI Mice

**FIGURE 1:**
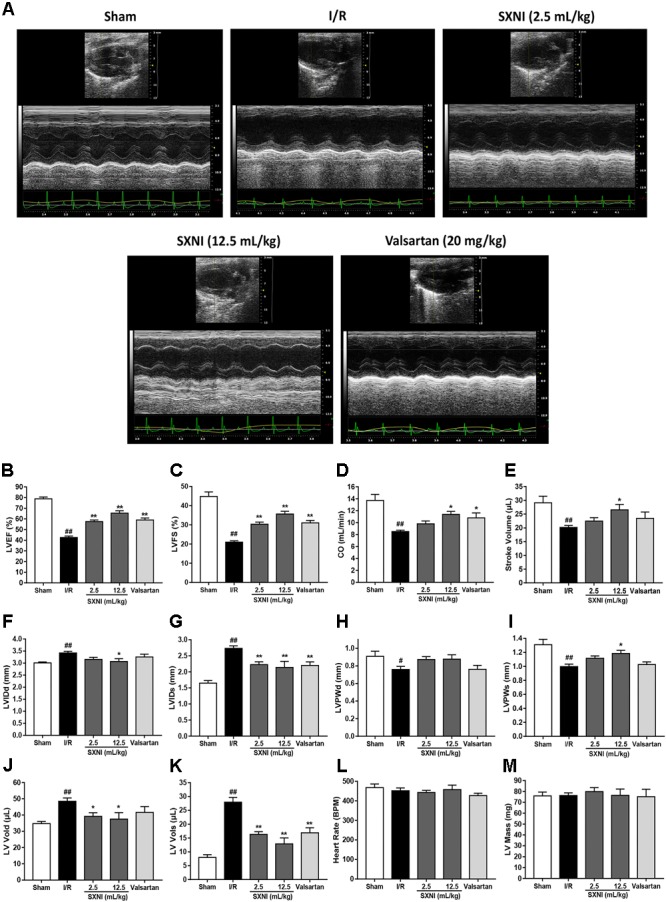
Echocardiographic evaluation of SXNI on cardiac function in MIRI mice. After 30 min of ischemia and 18 h of reperfusion, the curative effect of SXNI (2.5 mL/kg), SXNI (12.5 mL/kg), and valsartan (20 mg/kg) on cardiac function was evaluated. **(A)** Representative images of echocardiography. Cardiac performance was determined by echocardiography with different groups. **(B)** LVEF %, **(C)** LVFS %, **(D)** CO, **(E)** stroke volume, **(F)** LVIDd, **(G)** LVIDs, **(H)** LVPWd, **(I)** LVPWs, **(J)** LV Vold, **(K)** LV Vols, **(L)** heart rate, and **(M)** LV Mass were measured in M-mode. Results were presented as mean ± SEM (*n* = 7-10). ^#^*P* < 0.05, ^##^*P* < 0.01 vs. Sham group, ^∗^*P* < 0.05, ^∗∗^*P* < 0.01 vs. I/R group. (LV, left ventricular; EF, ejection fraction; FS, LV fractional shortening; CO, cardiac output; LVIDd, LV internal dimensions at diastole; LVIDs, LV internal diameter systole; LVPWd, LV posterior wall diastole; LVPWs, LV posterior wall systole; LV Vols, LV systole volume; LV Vold, LV diastole volume).

**FIGURE 2:**
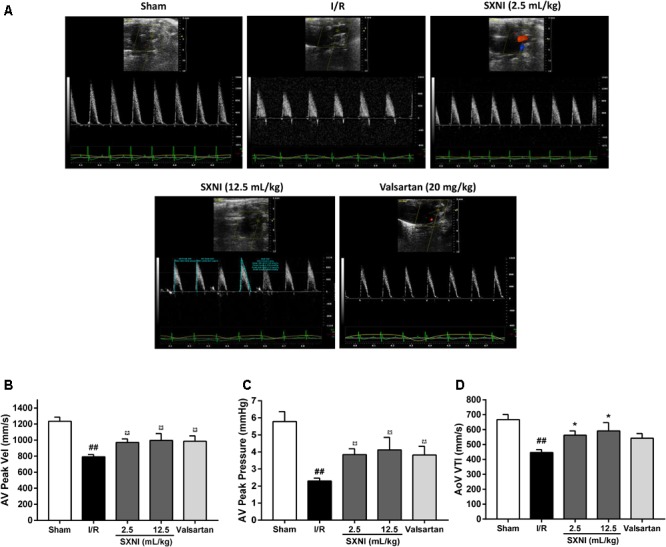
Effects of SXNI on coronary blood flow in MIRI mice. After 30 min of ischemia and 18 h of reperfusion, the curative effect of SXNI (2.5 and 12.5 mL/kg) and valsartan (20 mg/kg) on coronary blood flow was evaluated. **(A)** Representative echocardiography images of coronary blood flow were determined with different groups. **(B)** AV peak velocity, **(C)** AV peak pressure, and **(D)** AoV VTI were measured in Color Doppler mode. Results were presented as mean ± SEM (*n* = 7–10). ^#^*P* < 0.05, ^##^*P* < 0.01 vs. Sham group, ^∗^*P* < 0.05 vs. I/R group. (AV Peak Vel, aortic valve peak velocity; AoV VTI, aorta velocity-time integral mean velocity).

After 30 min of ischemia and 18 h of reperfusion, echocardiography was performed to determine the effects of SXNI on cardiac function. Compared to sham group, I/R group significantly decreased LVEF %, LVFS %, CO, stroke volume, LVPWs, and increased LVIDd, LVIDs, LV Vold, LV Vols, while it had no marked change in heart rate and LV Mass (**Figure 1**). By contrast, SXNI (2.5 and 12.5 mL/kg) and valsartan (20 mg/kg) markedly attenuated I/R-induced impairment of LVEF % (**Figure 1B**), LVFS % (**Figure 1C**), LVIDs (**Figure 1G**) and LV Vols (**Figure 1K**). SXNI at 12.5 mL/kg also improved the parameters of CO (**Figure 1D**), stroke volume (**Figure 1E**), LVIDd (**Figure 1F**), LVPWs (**Figure 1I**), and LV Vold (**Figure 1J**). Valsartan (20 mg/kg) also increased CO (**Figure 1D**). However, neither SXNI nor valsartan markedly affected LVPWd (**Figure 1H**), heart rate (**Figure 1L**), and LV Mass (**Figure 1M**) compared with the I/R group. Color images were acquired in color Doppler mode at 18 h after reperfusion to evaluate the effects of SXNI on coronary blood flow (**Figure 2A**). As expected, in the I/R group, AV peak velocity (**Figure 2B**), AV peak pressure (**Figure 2C**), and AoV VTI (**Figure 2D**) were significantly lower than those in the Sham group. However, these deficiencies were markedly improved by SXNI (2.5 and 12.5 mL/kg, **Figures 2B–D**) and valsartan (20 mg/kg) significantly increased AV peak velocity (**Figure 2B**) and AV peak pressure (**Figure 2C**). Collectively, these data suggest that SXNI treatment improves cardiac performance in the model of I/R injury.

### SXNI Attenuated Myocardial Injury in MIRI Mice

**FIGURE 3:**
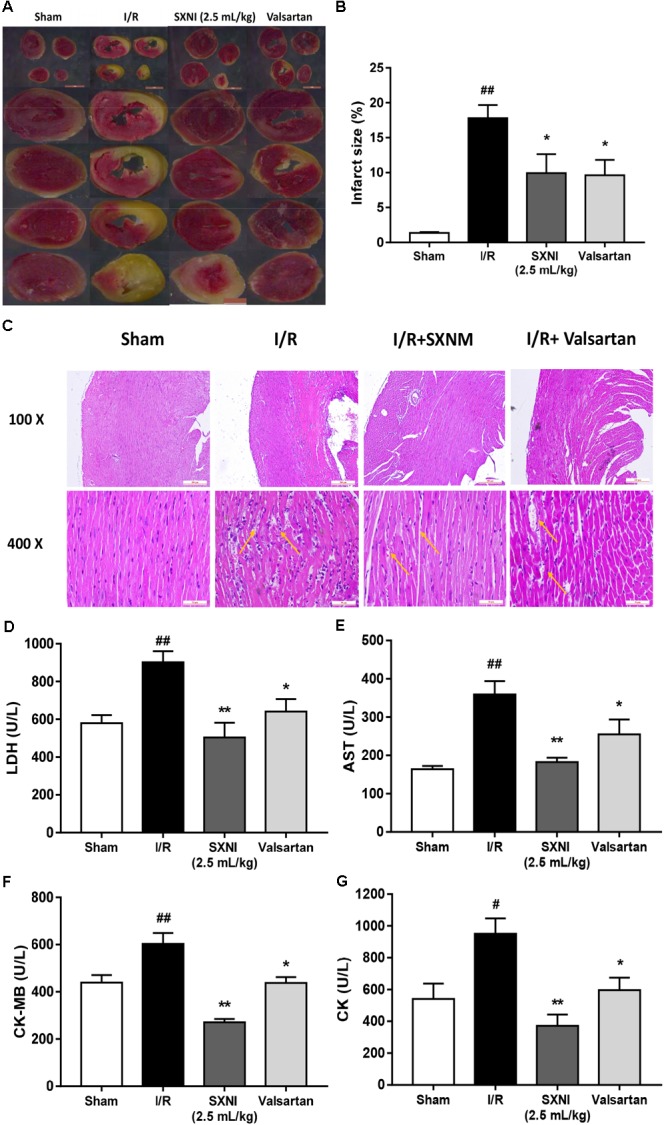
Effects of SXNI on myocardial injury in MIRI mice. After 30 min of ischemia and 24 h of reperfusion, the effect of SXNI on myocardial injury was evaluated. **(A)** TTC staining determined the effect of SXNI (2.5 mL/kg) and valsartan (20 mg/kg) on myocardial infarct area. **(B)** Bar graphic representation of myocardial infarct size (*n* = 4). **(C)** Representative H&E staining (100× and 400× magnification) pictures indicating the histopathological changes in I/R model as well as that caused by SXNI and valsartan. Yellow arrows indicated myocardium damage sections (*n* = 4). **(D–G)** The effect of SXNI and valsartan on release of LDH, AST, CK-MB, and CK in serum at the end of reperfusion (*n* = 6). Results were presented as mean ± SEM. ^#^*P* < 0.05, ^##^*P* < 0.01 vs. Sham group, ^∗^*P* < 0.05, ^∗∗^*P* < 0.01 vs. I/R group.

To investigate the effects of SXNI on MIRI, myocardial infarction size, histopathology and serum LDH, AST, CK-MB, and CK release were measured. After 30 min ischemia and 24 h reperfusion, the myocardial infarction size, morphology and serum LDH, AST, CK-MB, and CK activities were obviously altered compared with the sham group, resulting in myocardial injury (**Figure 3**). However, treatment groups significantly reduced myocardial infarct size (**Figures 3A,B**). Simultaneously, the histopathological examination of heart tissues from I/R group exhibited widespread myocardial structural disarray, increased necrosis and fusion area, and many inflammatory cells infiltrating the myocardial tissue, which were indicated with yellow arrows (**Figure 3C**). In contrast, the damaged histological features and cardiac structure were distinctly ameliorated after administration with SXNI (2.5 mL/kg) and valsartan (20 mg/kg) (**Figure 3C**). In contrast with I/R group, SXNI (2.5 mL/kg) and valsartan (20 mg/kg) markedly lowered the activities of LDH, AST, CK-MB, and CK in serum (**Figures 3D–G**).

### SXNI Decreased Infarction Volumes in CIRI Mice

**FIGURE 4:**
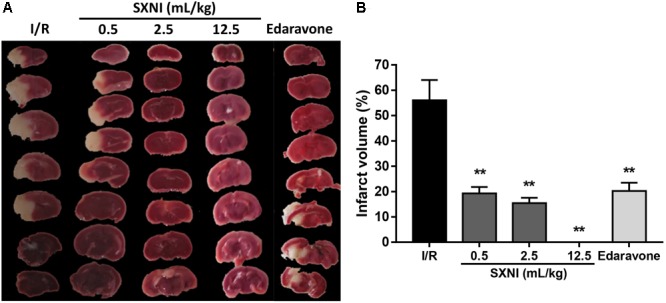
Effects of SXNI on cerebral injury in CIRI mice. After 90 min of ischemia and 24 h of reperfusion, the curative effect of SXNI on cerebral injury was evaluated. **(A)** Representative images of brain sections stained with TTC. After reperfusion for 24 h, five groups of mice were treated intravenously with saline (model), SXNI at 0.5, 2.5, and 12.5 mL/kg as well as edaravone (9 ml/kg), respectively. Ischemic infarctions (white area) were detected in all groups. **(B)** At 24 h after stroke, the percentage of infarct volumes was quantified (*n* = 6–8). Results were presented as mean ± SEM. ^∗∗^*P* < 0.01 vs. I/R group.

Cerebral ischemic-reperfusion caused significant cerebral infarct. At 24 h after initiation of ischemic stroke, SXNI at both 0.5 and 2.5 mL/kg significantly reduced infarct volumes caused by cerebral ischemic-reperfusion damage. Remarkably, SXNI at 12.5 mL/kg almost completely eliminated the cerebral infarction. Similar to SXNI, the cerebral infarction volumes were significantly reduced by edaravone, a neuroprotective agent used clinically and serving as a positive control (**Figures 4A,B**).

### Transcriptome Identification of DEGs in MIRI and CIRI Mice After SXNI Treatment

**FIGURE 5:**
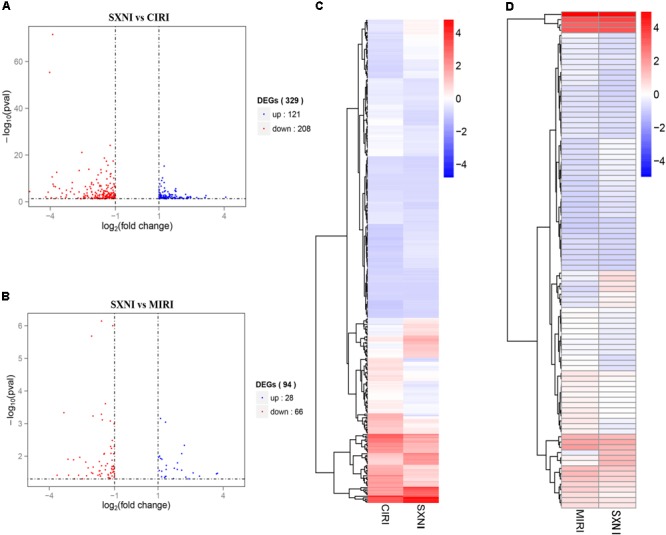
Differentially expressed gene (DEGs) of SXNI vs. CIRI and SXNI vs. MIRI. The overall distribution of differentially expressed genes (fold change ≥ 2 and *p*-Value ≤ 0.05) was reflected by the volcanic map. The *X*-axis represents the changes of gene expression in different samples and *Y*-axis represents the statistical significance of the difference in the gene expression. The up-regulated genes were in red and down-regulated genes in green. **(A)** The distribution of differently expressed genes between SXNI vs. CIRI samples, while **(B)** for the SXNI *vs.* MIRI samples. Hierarchical clustering representing the overall profiles of the transcripts that were significantly and differently expressed in **(C)** SXNI vs. CIRI samples, and **(D)** SXNI vs. MIRI samples.

To identify gene targets of SXNI in brain and heart, the left ventricular myocardium sections of MIRI and the brain slices from CIRI with or without SXNI treatment were subjected for high-throughput sequencing analyses. After mapping the sequencing reads to the reference genome, the relative expression levels of the transcripts were calculated in FPKM. Overall, there were 26,586 expressed genes in the CIRI +SXNI brain samples and 26,390 expressed genes in CIRI brain samples, whereas 24,444 expressed genes in the MIRI +SXNI heart samples and 24,347 expressed genes in the MIRI samples. Furthermore, 329 significantly altered DEGs with twofold or greater changes and *P*-value less than 0.05 were screened out in CIRI +SXNI samples over the CIRI samples. That included 121 upregulated and 208 downregulated genes (**Figure 5A**). In MIRI +SXNI samples over the MIRI samples, 94 significantly differential expressions, with 28 upregulated and 66 downregulated genes, were detected (**Figure 5B**). The details of the significantly DEGs are listed in **Supplementary Tables S1, S2** and the overall gene expression profiles of their hierarchical cluster are presented in **Figures 5C,D**.

### The Shared Pathways and Functions Between MIRI and CIRI After SXNI Treatment

**FIGURE 6:**
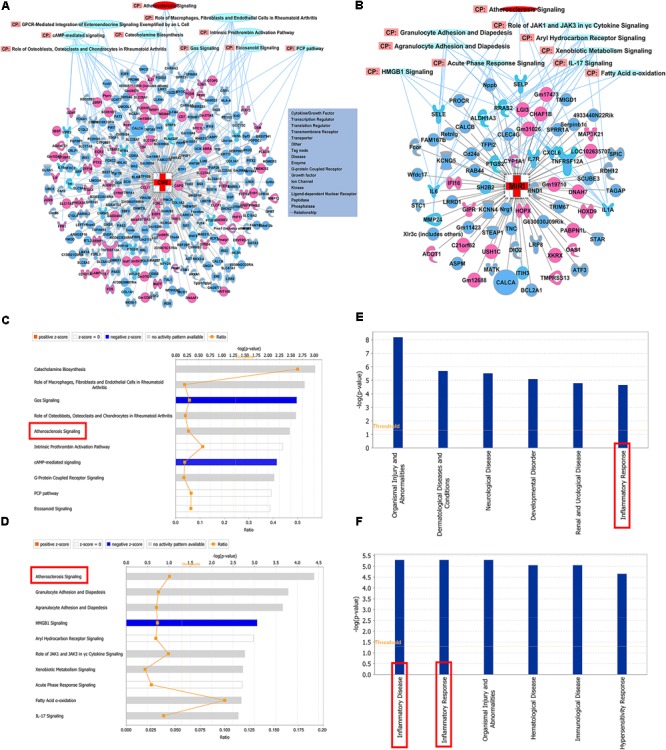
Shared mechanisms of SXNI on both MIRI and CIRI. DEGs with top 10 pathways in groups of **(A)** CIRI+SXNI vs. CIRI, and **(B)** MIRI+SXNI vs. MIRI (upregulated genes in red color, downregulated genes in blue and two amplifying common genes). The top 10 canonical pathways (CP) in groups of **(C)** CIRI+SXNI vs. CIRI, and **(D)** MIRI+SXNI vs. MIRI. Atherosclerosis signaling was shared in both groups. The top 6 functions in groups of **(E)** CIRI+SXNI vs. CIRI, and **(F)** MIRI+SXNI vs. MIRI. Inflammatory response was existed in both groups. The importance was ordered from top to bottom by –log (*p*-value).

**FIGURE 7:**
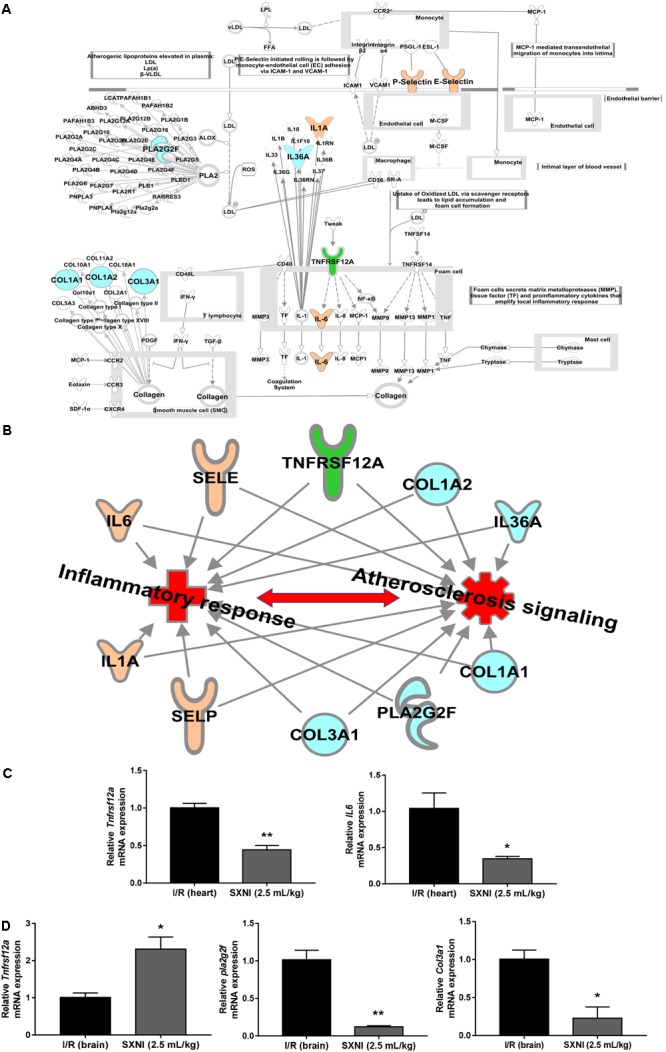
Validation of SXNI regulated genes in inflammatory and atherosclerosis signaling. **(A)** The details of atherosclerosis signaling with DEGs in two groups of SXNI vs. CIRI (blue) and SXNI vs. MIRI (yellow) were shown, *TNFRSF12A* was shared in both groups (green). **(B)** The relationship between inflammatory response and atherosclerosis signaling with the 10 genes was displayed. **(C**) *TNFRSF12A* and *IL6* in atherosclerosis signaling in heart were validated. **(D)** *TNFRSF12A, Pla2g2f*, and *Col3a1* in atherosclerosis signaling in brain were validated. Results were presented as mean ± SD, *n* = 3. ^∗^*P* < 0.05, ^∗∗^*P* < 0.01 vs. I/R group.

The significantly differentially expressed DEGs were submitted to IPA to execute core analysis. In separate groups of CIRI (**Figure 6A**) and MIRI (**Figure 6B**) after SXNI administration, upregulated (in red)- and downregulated (in blue)-genes and their corresponding top 10 pathways were displayed. Among the DEGs in both, two overlapping genes are detected between the brain tissues (SXNI vs. CIRI) and myocardium (SXNI vs. MIRI) (**Figure 2**), which are *Calca* (ENSMUSG00000030669) and *Tnfrsf12a* (ENSMUSG00000023905). *Calca* is associated with Parkinson diseases (Buervenich et al., 2001) and essential hypertension (Luo et al., 2008), while *Tnfrsf12a* is an important molecule in cardiac hypertrophy disease (Ma et al., 2016), cardiac shock (Wang W. et al., 2016) retinal inflammation (Abcouwer et al., 2013), and atherosclerosis-related diseases (Munoz-Garcia et al., 2006, 2011; Moreno et al., 2013; Fernandez-Laso et al., 2017). Top 10 canonical pathways and top 6 functions were listed by the descending -log(*p*-value) score (**Figures 6C–F**). The most impacted top 10 canonical signaling pathways with a descending order in brain were catecholamine biosynthesis, role of macrophages, fibroblasts and endothelial cells in rheumatoid arthritis, gas signaling, role of osteoblasts, osteoclasts and chondrocytes in rheumatoid arthritis, atherosclerosis signaling, intrinsic prothrombin activation pathway, cAMP-mediated signaling, G-protein coupled receptor signaling, PCP pathway and eicosanoid signaling. The most related top 10 canonical signaling pathways in turn in heart were atherosclerosis signaling, granulocyte adhesion and diapedesis, agranulocyte adhesion and diapedesis, HMGB1 signaling, aryl hydrocarbon receptor signaling, role of JAK1 and JAK3 in γc cytokine signaling, xenobiotic metabolism signaling, acute phase response signaling, fatty acid α-oxidation and IL-17 signaling. Notably, the pathway of atherosclerosis signaling and the function of inflammatory response were affected in both I/R brain tissues and myocardium (**Figures 6C–F**), indicating that they might serve as a common mechanism in response to SXNI treatment for both CIRI and MIRI. Detailed depiction of AS signaling based on the gene expression data was shown in **Figure 7A** (brain genes in blue, heart genes in yellow and both in green). These genes included *Pla2g2f, Il1f9, Col1a1, Col1a2*, and *Col3a1* in the brain tissues (in yellow), while *SELP, SELE, IL6* and *IL1A* were identified in the myocardium (in blue), and *Tnfrsf12a* in both (in green). Interestingly, these 10 highly regulated genes by SXNI were also relevant to inflammatory response. The relationship between inflammatory response and atherosclerosis signaling with the 10 genes in separate groups was shown (**Figure 7B**). Collectively, they provide new molecular insights into the known notion that active ingredients of Ginkgo biloba extract in SXNI play an important role in protecting against cardio- and cerebral vascular diseases.

### Validation of DEGs in AS Signaling

Aiming to validate the results of transcriptome analysis, five genes in AS signaling, including *Tnfrsf12a, IL-6* in hearts (**Figure 7C**) and *Tnfrsf12a, Pla2g2f*, and *Col3a1* in brains (**Figure 7D**), were extracted for qPCR validation. The qPCR results were consistent with the transcriptome data, suggesting the transcriptome data were reliable. Of note, identical to the transcriptome results, *Tnfrsf12a* expression in I/R heart was downregulated 55.6% (**Figure 7C**) whereas in I/R brain was upregulated 130.9% (**Figure 7D**) by SXNI, respectively.

## Discussion

There are several new discoveries in this study: (1) this is the first report that SXNI, another EGB, had a protective effect on both CIRI-induced cerebral infarction and MIRI-induced myocardial infarction and cardiac dysfunction; (2) RNA-seq analysis identified 329 DEGs in SXNI vs. CIRI and 94 DEGs in SXNI vs. MIRI, with two shared genes of *TNFRSF12A* and *Calca*; (3) IPA analysis revealed top 10 canonical signaling pathways including catecholamine biosynthesis, atherosclerosis signaling, cAMP-mediated signaling, G-protein coupled receptor signaling and eicosanoid signaling, and top 6 functions such as organismal injury and abnormalities, neurological disease and inflammatory response in SXNI vs. CIRI, whereas top 10 canonical signaling pathway including atherosclerosis signaling, HMGB1 signaling, aryl hydrocarbon receptor signaling and fatty acid α-oxidation, and top 6 functions including inflammatory disease, inflammatory response and immunological disease in SXNI vs. MIRI; (4) atherosclerosis signaling and inflammatory response merged as a common theme in both by comparison. Ten key regulated genes (including *Pla2g2f, Il1f9, Col1a1, Col1a2*, and *Col3a1* in the brain, while *SELP, SELE, IL6*, and *IL1A* in the myocardium, and *Tnfrsf12a* in both) are correlative with atherosclerosis signaling and inflammatory response.

Inflammatory response is by now well recognized as a critical contributor for the initiation and development of atherosclerotic cardiovascular disease (Libby et al., 2002), and contributes to ischemic cardiovascular disease linking stroke to cardiac dysfunction (Libby et al., 2016; Chen Z. et al., 2017). Two recent studies reported that canakinumab targeting the interleukin-1β (IL-1β) immunity pathway was regarded as new anti-inflammatory strategies to reduce atherosclerotic disease (Ridker et al., 2017a) and incident lung cancer with atherosclerosis in patient (Ridker et al., 2017b), proving that anti-inflammatory therapy is a valid curative method to lower cardiovascular events. These examples directly support the notion that inflammatory response is capable of serving as the common mechanism for SXNI to treat both CIRI and MIRI. In addition, atherosclerosis signaling, another common mechanism of SXNI action, is recorded as a canonical pathway in IPA under the pathway category of cardiovascular signaling and disease-specific pathways, indicating that SXNI may exert an effect on atherosclerosis by targeting the ten identified genes, which need to be experimentally verified in future studies.

It is worth noticing that the ten SXNI-regulated genes in atherosclerosis signaling are also related to inflammatory response, especially the *Tnfrsf12a* gene, which was shared in both brain and heart tissues. Also called as fibroblast growth factor-inducible 14 (Fn14), *Tnfrsf12a* is the smallest but most powerful TNFR superfamily member described so far, which is a plasma membrane receptor of TNF-like weak inducer of apoptosis (TWEAK). TWEAK and/or *Tnfrsf12a* has been identified as potential therapeutic targets in numerous diseases, including (1) cancers such as (Winkles et al., 2006) cancer-induced cachexia (Johnston et al., 2015), glioblastoma (Perez et al., 2016), melanoma (Zhou et al., 2013), gastric cancer (Kwon et al., 2012), ovarian cancer (Dai et al., 2009), triple-negative breast cancer (Zhou et al., 2014), and esophageal adenocarcinoma (Watts et al., 2007); (2) liver diseases such as alcoholic hepatitis (Affo et al., 2013) and non-alcoholic fatty liver disease (NAFLD) (Lozano-Bartolomé et al., 2016); (3) kidney diseases such as renal ischemia reperfusion injury (Hotta et al., 2011), fibrotic kidney disease (Xia et al., 2015; Wilhelm et al., 2016) and kidney injury (Ortiz et al., 2009; Sanz et al., 2011; Weinberg, 2011); (4) and a variety of other diseases such as graft- vs. -host disease (GVHD) (Chopra et al., 2015; MacEwan, 2015), atopic dermatitis (AD) and psoriasis (Sidler et al., 2017), acute intestinal inflammation (Di Martino et al., 2016), neuropsychiatric disease (Wen et al., 2013), lupus nephritis (Michaelson et al., 2012), bullous pemphigoid (Liu et al., 2017), cutaneous disease (Doerner et al., 2015), chronic colitis (Son et al., 2013), amyotrophic lateral sclerosis (Bowerman et al., 2015), myotonic dystrophy (Yadava et al., 2015). Importantly, *Tnfrsf12a* receptor protects against right heart fibrosis and dysfunction (Novoyatleva et al., 2013) and cardiomyocyte proliferation (Novoyatleva et al., 2010) in heart-related diseases. On the other hand, *Tnfrsf12a* expression in brain is regarded as a prognostic/predictive biomarker of brain metastasis (Martinez-Aranda et al., 2015), and protects neurodegeneration (Potrovita et al., 2004) and cerebral ischemia (Zhang et al., 2007). Interestingly, our results showed that *Tnfrsf12a* was upregulated in SXNI vs. CIRI group, while downregulated in SXNI vs. CIRI group, remarkably coincident with the detrimental or beneficial functions of this gene in heart and brain, respectively. This may also attribute to the bidirectional effect of *Tnfrsf12a*, such as pro-inflammatory (Campbell et al., 2006) and anti-inflammatory effect (Sidler et al., 2017). In certain instances of acute injury, *Tnfrsf12a* signaling would be transient in nature and beneficial. However, in conditions of chronic tissue injury, *Tnfrsf12a* expressed in high levels could result in harmful, pathological effects (Winkles, 2008). In addition, for one thing, mice lacking *Tnfrsf12a* substantially reduced right ventricular fibrosis and dysfunction (Novoyatleva et al., 2013). For another, deletion of *Tnfrsf12a* promotes blood brain barrier (BBB) disruption and increases neuronal cell death (Wen et al., 2015). Taken together, *Tnfrsf12a* may serve as a common therapeutic target for SXNI to treat both CIRI and MIRI, which should be further elucidated in the future.

In addition to the shared inflammation-atherosclerosis signaling pathways in I/R brain and heart by SXNI discussed above, our transcriptome analysis also indicated a number of interesting unique targeting genes/pathways in each tissue. For example, catecholamine biosynthesis and organismal injury and abnormalities are ranked as number one disease and function in I/R brain treated with SXNI, respectively (**Figures 6C,E**). Inhibition of catecholamine biosynthesis has been shown to reduce acute ischemic stroke (Welch et al., 1977) and this may provide a new light for SXNI to treat CIRI. Protection of brain from injury and related symptoms are the most documented beneficial effects by EGB (Calapai et al., 2000; DeKosky et al., 2008; Oliveira et al., 2009; Shi et al., 2010; Rocher et al., 2011; Abdel-Wahab and Abd El-Aziz, 2012; Tanaka et al., 2013; Tulsulkar and Shah, 2013; Nada et al., 2014; Belviranli and Okudan, 2015; Liu et al., 2015; Wu et al., 2015; Kim et al., 2016; Tulsulkar et al., 2016). G-protein coupled receptor (GPCR) are the most abundant receptor type in the central nervous system and are linked to platelet activation, BBB function and endothelial dysfunction (Guerram et al., 2016), leading to another insight of SXNI for CIRI. HMGB1 is a danger signal that senses cell damage especially in cardiac ischemia, and is an important candidate biomarker predicting the risk of cardiovascular events in clinic (Peter and Bobik, 2012). Besides, aryl hydrocarbon receptor signaling plays a key role in myocardium reperfusion-triggered stress response (Vilahur et al., 2013). SXNI effects on HMGB1 signaling and aryl hydrocarbon receptor signaling, particularly regarding the MIRI, should be paid more attention in further studies.

Although SXNI could significantly protect both CIRI and MIRI in our experiments, it is still possible that it is more inclined to target CIRI than MIRI, as more transcriptome DEGs were identified in SXNI vs. CIRI than SXNI vs. MIRI. SXNI at high dose even reversed the brain damage completely, consistent with the fact that more EGB-related beneficial reports are on brain and to a much less extent, on the heart. In previous studies, EGB was well documented to treat cerebral- and myocardial ischemia, and SXNI, a clinically proven prescription of EGB, provides a good model to further explore the major active ingredients. Our ongoing study on identification of the main ingredients (mainly derived from flavonols and terpene trilactones) and their selective pharmacological action on brain and heart should facilitate a deeper understanding of the brain-heart protection mechanisms of EGB in the future.

## Conclusion

Our RNA-seq and network pharmacology analyses revealed for the first time that SXNI ameliorates both CIRI-induced cerebral infarction and MIRI-induced myocardial infarction. The common mechanisms shared in both are linked with atherosclerosis signaling and inflammatory response with at least the following target genes: *Tnfrsf12a, Pla2g2f, Il1f9, Col1a1, Col1a2*, and *Col3a1* in brain, while *Tnfrsf12a, SELP, SELE, IL6*, and *IL1A* in myocardium. Besides, the opposite regulation of *Tnfrsf12a* by SXNI in CIRI and MIRI is consistent with the documented function of this gene in the respective tissues. Overall, this study provides a new understanding of SXNI in clinical application on I/R-induced cardio- and cerebral vascular diseases.

## Author Contributions

YZ conceived and organized the study. ML performed the myocardial ischemia-perfusion correlative experiments and prepared **Figures 1–3**. TZ performed the cerebral ischemia-perfusion assay and drew **Figure 4**. YC and ML performed the transcriptome and IPA analysis and depicted **Figures 5–7**. ZN, JR, and XJ participated in the experiments. GF helped with the design of the study and interpretation of results. YZ, ML, YC, and TZ wrote the manuscript. All authors reviewed and approved the manuscript.

## Conflict of Interest Statement

The authors declare that the research was conducted in the absence of any commercial or financial relationships that could be construed as a potential conflict of interest. The reviewer ID and handling Editor declared their shared affiliation.

## Supplementary Material

TABLE S1 Total 329 significantly differentially expressed genes in SXNI vs. CIRI.

TABLE S2 Total 94 significantly differentially expressed genes in SXNI and MIRI.
